# Multisystemic Assessment in Andersen–Tawil Syndrome: Report of Eighteen Individuals

**DOI:** 10.3390/diagnostics16121876

**Published:** 2026-06-16

**Authors:** Maria Gnazzo, Giovanni Parlapiano, Silvia Morlino, Mafalda Mucciolo, Daniele Minervino, Marco Castori, Francesca Mercadante, Michele Trifiletti, Maria Gabriela Obregon, Paolo Prontera, Daniela Righi, Francesca Di Lorenzo, Massimo Stefano Silvetti, Maria Lisa Dentici, Andrea Bartuli, Fabrizio Drago, Antonio Novelli, Anwar Baban

**Affiliations:** 1 Laboratory of Medical Genetics, Translational Cytogenomics Research Unit, Bambino Gesù Children’s Hospital, IRCCS, 00165 Rome, Italy; maria.gnazzo@opbg.net (M.G.);; 2 Cardiogenetic Center, Rare Diseases and Medical Genetics Units, Bambino Gesù Children’s Hospital, IRCCS, 00165 Rome, Italy; 3 Division of Medical Genetics, Fondazione Casa Sollievo della Sofferenza, IRCCS, 71013 San Giovanni Rotondo, Italy; 4 Medical Genetics Unit, Villa Sofia-Cervello Hospitals, 90146 Palermo, Italy; 5 Department of Social Sciences, Universidad Católica San Antonio de Murcia (UCAM), 30107 Murcia, Spain; mtrifiletti@alu.ucam.edu; 6 Genetics Department, Hospital Garrahan, Buenos Aires C1245, Argentina; 7 Medical Genetics Unit, S. Maria della Misericordia Hospital, 06129 Perugia, Italy; 8 Pediatric Cardiology and Arrhythmia/Syncope Complex Unit, Bambino Gesù Children’s Hospital, IRCCS, 00165 Rome, Italy; 9 Rare Diseases and Medical Genetics Units, Bambino Gesù Children Hospital and Research Institute, IRCCS, 00165 Rome, Italy; 10 The European Reference Network for Rare, Low Prevalence and Complex Diseases of the Heart (ERN GUARD-Heart), 1105 Amsterdam, The Netherlands; 11 UniCamillus-Saint Camillus International University of Health Sciences, 00131 Rome, Italy; 12 Mohammed Bin Rashid University of Medicine and Health Sciences (MBRU), Mediclinic Hospitals MCME, Dubai P.O. Box 505055, United Arab Emirates

**Keywords:** Andersen–Tawil Syndrome, Long QT, periodic paralysis, multisystemic assessment

## Abstract

**Background/Objectives:** Andersen–Tawil Syndrome (ATS) is an ultra-rare autosomal dominant condition secondary to deleterious variants in *KCNJ2* or *KCNJ5* in the majority of patients. It is variably characterized by a triad of Long QT Syndrome (LQTS)/ventricular arrhythmias with a prominent U-wave, episodic flaccid muscle weakness/paralysis and skeletal abnormalities. Other clinical features include distinctive facial dysmorphisms, dental anomalies, and mild learning difficulties. Limited data are available regarding the initial presenting sign or symptoms of ATS. **Methods:** In this study, we include data from 18 patients across eight families. In our cohort, the main clues that led probands to genetic testing were syncope (three families), which was associated with dysmorphic features in one case; LQTS (one family); asymptomatic premature ventricular contractions (PVCs) (three families); and a case incidentally identified during routine cardiac evaluations and due to short stature (one family). **Results:** Following thorough investigations, a prolonged QT interval was detected in five individuals and prominent U-waves were observed in the majority of the court. Distinctive facial features were consistently present (100%) and can be suggested as a clinical tool for accelerated diagnosis. Skeletal manifestations ranged from 37.5% to 93.7% including short stature, scoliosis and finger defects. Only two patients showed periodic paralysis (PP). **Conclusions:** Regarding the clinical management of ATS, we underline the importance of the multidisciplinary, personalized, and longitudinal approach, where arrhythmia may not be the leading sign but remains the most potentially critical prognostic factor.

## 1. Introduction

Andersen–Tawil Syndrome (ATS) is a very rare multisystem channelopathy characterized by an extremely variable phenotype ranging from cardiac to extra-cardiac features. The estimated prevalence in the population is around 0.8–1.06 in 1,000,000 [1,2]. Clinical presentation includes variable degrees of involvement of the triad:

(a) Cardiac manifestations of a potential progressive nature: prolonged QTc or QUc intervals, prominent U-waves, bidirectional ventricular tachycardia (VT), premature ventricular contractions (PVCs), and, rarely, polymorphic VT or torsade de pointes.
(b) Muscular manifestations: episodic flaccid muscle weakness, painful symptoms and periodic paralysis (PP).
(c) Skeletal/craniofacial manifestations with peculiar dysmorphic features (broad forehead, triangular face, maxillary and mandibular hypoplasia, low-set ears, short palpebral fissures, wide nasal bridge, bulbous nose, thin upper lip, arched palate, oligodontia and dental crowding), hand and foot abnormalities (fifth finger clinodactyly, second–third toe syndactyly, brachydactyly), scoliosis, short stature, and joint laxity [3].

The clinical triad “heart–muscle–skeleton/craniofacial” was reported to range from in 42.9% to 60% of patients [4,5,6,7]. In the remaining patients, it is possible to detect just one or two of the major involvements.

Some patients may also show intellectual disability with mild learning difficulties and abnormalities of executive function and abstract reasoning.

The proposed criteria for ATS suggest a clinical diagnosis if two of these four criteria are detected: (1) periodic paralysis; (2) ventricular arrhythmias, prolonged QTc or QUc interval, and/or a prominent U wave; (3) at least two of the following dysmorphic features: low-set ears, wide-set eyes, small mandible, fifth digit clinodactyly, syndactyly and short stature; (4) a family member with confirmed ATS [8].

From the genetic point of view, ATS is an autosomal dominant condition caused in 50–60% of the cases by likely pathogenic (LP) or pathogenic (P) variants in *KCNJ2* (Gene/Locus MIM number: 600681, hyperlink “https://omim.org/entry/600681, accessed on 8 January 2026) encoding the α subunit of the K+ channel protein Kir2.1 [9,10]. *KCNJ2*-related ATS defines Type 1. About 15% of patients present LP or P variants in *KCNJ5* (Gene/Locus MIM number: 600734, hyperlink https://omim.org/entry/600734, accessed on 8 January 2026) encoding the G protein-sensitive-activated inwardly rectifying K+ channel Kir3.4 [5]. *KCNJ5*-related ATS defines Type 2. About 30–40% of cases are genetically elusive, suggesting the presence of other possible unknown causative mechanisms/genes.

The aim of this study is to investigate and describe the clinical implications of ATS, focusing not only on the cardiac spectrum but also on multisystemic manifestations.

## 2. Materials and Methods

**Figure 1:**
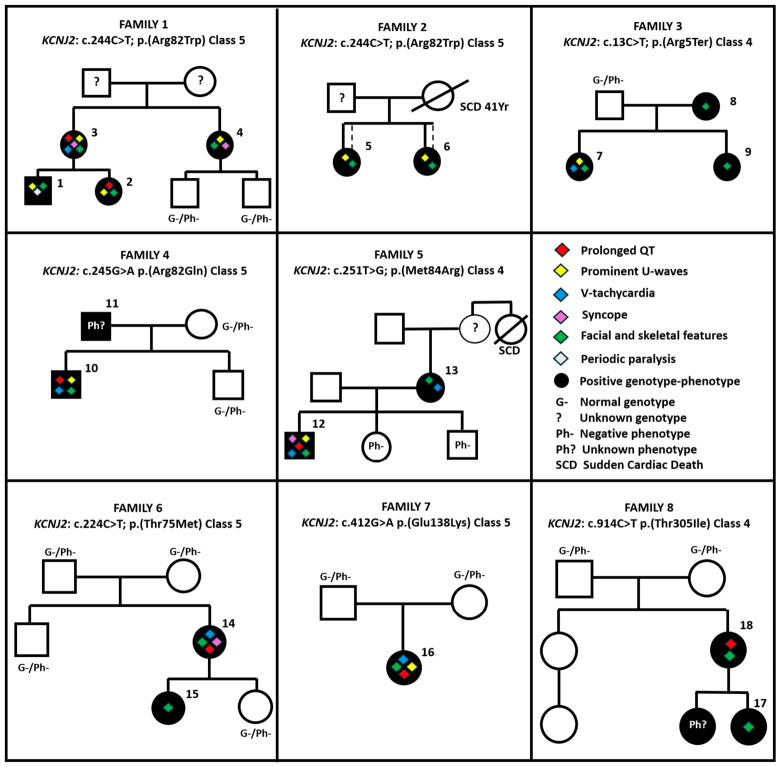
Family Pedigrees.

We describe 18 patients from 8 unrelated families with genetically confirmed ATS related to 7 *KCNJ2* variants. Figure 1 includes pedigrees with major clinical features.

Genetic Analysis Method: Clinical Exome Sequencing (CES) was performed on genomic DNA, extracted from peripheral blood leukocytes using the ClinEX pro kit (4bases, Manno, Switzerland) according to the manufacturer’s protocol and sequenced on the Illumina NovaSeq 6000 platform (Illumina, San Diego, CA, USA). The FASTQ files were aligned to the human reference genome GRCh37/hg19. The DRAGEN Enrichment v4.0.3 application (Illumina, Inc.) and GENEYX analysis (LifeMap Sciences, Herzliya, Israel) were used for calling and annotating variants, respectively. Variant filtering retained only high-quality, coding/splice-site variants, with an allele frequency ≤ 0.1% based on the Genome Aggregation Database (gnomAD v2.1.1). The deleterious impact of the variants was scored with Combined Annotation Dependent Depletion (CADD) v1.6 and the Rare Exome Variant Ensemble Learner (REVEL, version data: 3 June 2021). Selected variants were interpreted according to the American College of Medical Genetics and Genomics (ACMG) guidelines. Variants were described on the *KCNJ2* NM_000891.3 RefSeq transcript using HGVS recommendations.

Pediatric patients were included in a specific and personalized follow-up program. Multisystemic screening and surveillance were established focusing on ATS manifestations, including the arrhythmic spectrum and multiorgan features such as muscular and skeletal involvement. Data collection included family history, prenatal findings, perinatal parameters, major developmental milestones, electrocardiograms (ECG), 24 h ECG Holter monitoring, cardiac stress tests, echocardiography, reports of clinical symptoms such as palpitations or muscle weakness, growth parameters, dysmorphic features, renal, liver and thyroid function tests, electrolyte measurements, endocrine data, and occupational, orthopedic, ophthalmological, and psychological evaluations.

Adult patients were referred to specific cardiac centers, and data relating to their multisystem assessments were collected.

### 2.1. Family 1

The index case is a 46-year-old Italian female patient with recurrent syncope for 8 years, despite normal baseline cardiac evaluations (ECG, echocardiography and cardiac MRI). She reported an attack of muscle weakness during her teenage years after physical exercise with spontaneous resolution. After repeated syncope (more than 5 occasions within a short time), at 25 years of age, ECG showed polymorphic PVCs in couples and triplets; an endocavitary electrophysiological study (EPS) was negative for VT. At 37 years, she had a VT episode during a stress test. An implantable loop recorder (ILR) was considered after a syncopal episode while swimming in the sea. It reported both tachyarrhythmia and bradyarrhythmias. At 39 years old, her ECG showed prolonged QTc interval (470–480 ms) and prominent U-waves in V3–V6; at that time, she received an ICD implantation. At 40 years, she had two more syncopal episodes; in one of these, she was swimming. She also reported subluxations and articular sprain episodes related to joint hypermobility. Physical evaluation showed short stature, an asymmetric triangular face, low-set ears, prominent ear tips, temporomandibular joint instability, hand and foot brachydactyly, fifth digit clinodactyly, piezogenic papules, scoliosis, soft skin and joint laxity. NGS custom panel showed the pathogenic variant *KCNJ2*: c.244C>T; p.(Arg82Trp). Height was 151 cm (<3rd percentile).

Familial studies detected the same variant in her sister and in both of her children.

The 44-year-old sister had a negative cardiac assessment with a normal ECG and 24 h Holter ECG. Her clinical history was positive for one syncopal episode and negative for muscle disorders. She presented with short stature, triangular face, low-set ears, maxillary hypoplasia, hands and feet brachydactyly, fifth digit clinodactyly and scoliosis. Height was 150.5 cm (<3rd percentile).

**Figure 2:**
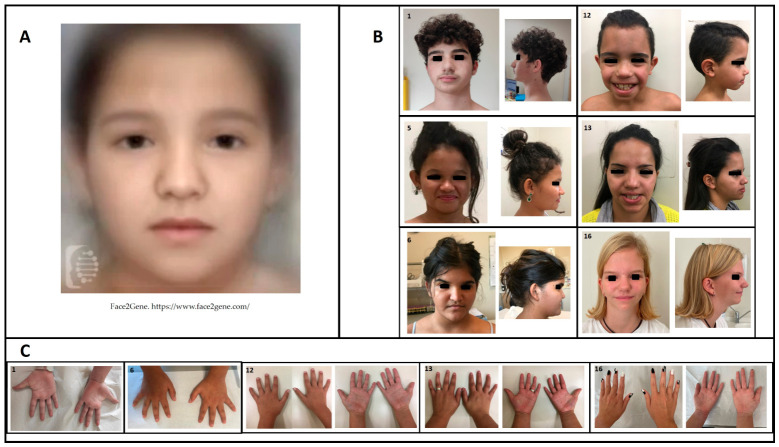
Distinctive facial features reported in the Gene2Face database (**A**). Facial features (**B**) and hands (**C**) from selected patients with *KCNJ2 deleterious* variants.

The 14-year-old son, who had an uneventful perinatal history, had a birth weight, length and occipitofrontal circumference (OFC) of 2.830 kg, 48 cm and 34.5 cm, respectively. He showed normal growth and appropriately reached developmental milestones. Cardiac screening, performed after the maternal ATS diagnosis, showed prominent U-waves in V2 and a normal QTc interval. Dysmorphic evaluation showed typical ATS facial features with low-set ears and short palpebral fissures; musculoskeletal examination detected hands and feet brachydactyly, fifth digit clinodactyly, syndactyly, lordosis, scoliosis and *cutis-marmorata*-like skin (Figure 2B,C, Pt 1). The patient required a 4-day hospitalization for prolonged neuromuscular paralysis during hypokalemia. At age 14, his height was 164 cm (50th percentile).

The 12-year-old daughter had an uneventful perinatal history; her birth weight, length and OFC were 3.350 kg, 50 cm and 34.5 cm, respectively. She showed normal growth and appropriately reached developmental milestones. She had an ECG at birth screening with a QTc value of 460 ms, which was never detected at or beyond that limit afterwards. Subsequent ECGs reported a normal QTc but revealed a prominent U-wave. She also presented ATS facial and skeletal traits such as a triangular face, low-set ears, brachydactyly, syndactyly, fifth digit clinodactyly and scoliosis. At age 12, her height was 153 cm (25th–50th percentile).

None of the Family 1 members presented neurocognitive disorders. After the ATS diagnosis, the two adult patients started Bisoprolol treatment, and the two pediatric patients were prescribed Nadolol.

### 2.2. Family 2

Family 2 is represented by two adopted sisters aged 17 and 15, originally from Colombia, who were referred to the endocrinology unit for short stature. Limited data were available regarding their family history, except for the sudden cardiac death (SCD) of their mother at the age of 41 years.

Both sisters showed a similar facial (Figure 2B, Pt 5,6) and skeletal phenotype characterized by small low-set ears, sunken eyes, mild hypertelorism, high arched palate, hands and feet brachydactyly, fifth digit clinodactyly, mild scoliosis, lordosis, small feet, second and third digit foot syndactyly, *pes planus*, piezogenic papules and joint laxity (Beighton score > 7/9) (Figure 2C, Pt 6). They presented mild neurocognitive impairment with learning difficulties; the younger sister also had oppositive traits. At the age of 17, the older sister’s height was 137.5 cm (<3rd percentile), and, at the age of 15, the younger sister’s height was 140 cm (<3rd percentile).

The short stature NGS custom panel showed the same pathogenic variant, *KCNJ2*: c.244C>T; p.(Arg82Trp), in both patients.

Considering the ATS diagnosis, the patients received multisystemic assessments; the cardiac evaluation revealed a normal QTc interval and a prominent U-wave. They subsequently started nadolol medication.

### 2.3. Family 3

The index case is a 19-year-old Italian girl who has been in a sports medicine follow-up program since she was 10 years of age for PVCs. She had an uneventful perinatal history; birth weight was 2.540 kg. She showed normal growth and appropriately reached developmental milestones. No symptoms were reported in her clinical history in spite of competitive swimming activities for almost 8 years. Cardiac magnetic resonance imaging (MRI) was normal. At 18 years old, during a stress test and 24 h ECG Holter test, she presented bidirectional VT, polymorphic PVCs, in couples and triplets, ventricular bigeminy, frequent non-sustained VT and a prominent U-wave. Physical assessment showed short stature, broad forehead, down-slanting palpebral fissures, multiple dental caries, a low-set right ear, mandibular hypoplasia, hand brachydactyly and camptodactyly, fifth digit clinodactyly, *genu valgum*, small feet with brachydactyly, and second, third and fourth digit toe syndactyly. At age 19, her height was 151 cm (<3rd percentile). She started Nadolol and Flecainide medication.

A channelopathy trio-based NGS custom panel revealed the likely pathogenic variant *KCNJ2*: c.13C>T; p.(Arg5Ter), which was maternally inherited.

The mother presented with the classic ATS phenotype with short stature, dysmorphic facial features and finger defects (hand and foot brachydactyly, fifth digit clinodactyly, second and third digit foot syndactyly).

Familial studies detected the same variant in her sister, who also showed distinctive facial and skeletal features with a final height at age 24 of 150.5 cm (<3rd).

Normal ECGs were reported for both relatives, but these were not directly reviewed by our group.

### 2.4. Family 4

The index case is a 20-year-old Italian boy. At 12 years of age, he presented with complex PVCs during ECG. Extensive cardiac screening showed numerous polymorphic PVCs (arrhythmic burden of 50%) in couples, as well as low-frequency, non-sustained VT runs, ventricular bigeminy, and QTc prolongation (490 ms) during a cardiac stress test. An electrophysiological (EP) study was negative for tachyarrhythmia inducibility. Echocardiography also detected a globular and slightly dilated left ventricle, with ejection fraction (EF) ranging from 45% to 50%, along with mild aortic root dilatation with minimal mitral and aortic insufficiency. Cardiac MRI confirmed mild left ventricular dilatation with moderate systolic dysfunction (EF 45%). He started nadolol and enalapril medication. While on therapy, a 24 h Holter ECG revealed sinus bradycardia, QUc prolongation, and prominent U-waves (fused with the preceding T and subsequent P waves), in the absence of ventricular bigeminy. During subsequent cardiac follow-up, left ventricular EF increased to 58%, and enalapril treatment was discontinued after 18 months.

A channelopathy NGS custom panel detected the pathogenic variant *KCNJ2*: c.245G>A p.(Arg82Gln), which was paternally inherited.

Antiarrhythmic therapy was optimized with a nadolol–flecainide combination, resulting in a significant reduction in PVCs and overall arrhythmic burden. The patient had normal height; at a skeletal level, he presented *pectus excavatum*. At age 20, his height was 180 cm (50th–75th percentile).

Limited information was available regarding the father’s clinical history; however, no cardiac or muscular symptoms were reported.

### 2.5. Family 5

The index case is a 15-year-old Argentine boy who has been in a Genetics and Cardiology follow-up program since the age of 8 due to arrhythmia and dysmorphism. He experienced a syncopal episode lasting three minutes following a knee trauma. ECG detected polymorphic PVCs with non-sustained VT. An echocardiogram showed mild left ventricular dilatation with normal ejection fraction.

Physical assessment revealed broad forehead, telecanthus, upturned nose, smooth philtrum, thin upper lip, high-arched palate, taurodontism with tooth malposition, retrognathia, dimple chin, ears with prominent anti-helix, brachydactyly, non-unique palmar transverse crease of left hand, and the distal interdigital crease of the second finger erased (Figure 2B,C, Pt 12). He had normal intellectual development.

A channelopathy trio-based NGS custom panel revealed the likely pathogenic variant *KCNJ2*: c.251T>G p.(Met84Arg), maternal-inherited.

The mother presented with short stature and classic ATS dysmorphic features including a wide forehead, telecanthus, upturned nose, modeled philtrum and large teeth with malposition. Her hands showed brachydactyly, tapering fingers, bilateral non-unique palmar transverse crease, and effacement of distal interdigital folds (Figure 2B,C, Pt 13). A 24 h Holter ECG detected polymorphic PVCs with non-sustained VT episodes. She was treated with amiodarone.

### 2.6. Family 6

The index case is a 36-year- old Italian woman with a history of ventricular bigeminy from the age of 10.

At 35 years old, she was admitted to the cardiology unit due to syncope with a non-sustained VT revealed. Holter ECG monitoring showed non-sustained VT and polymorphic PVCs. A stress test confirmed PVCs, including ones in premature ventricular couplets and triplets. Echocardiography and cardiac MRI showed normal cardiac anatomy. Therefore, she was initially discharged with a diagnosis of catecholaminergic polymorphic ventricular tachycardia.

Physical assessment showed short stature, short and downslanting palpebral fissures, a wide bridge and bulbous tip of the nose, thin upper lip, and fifth digit clinodactyly. She also had a history of learning disability.

A channelopathy NGS custom panel revealed the pathogenic de novo variant *KCNJ2*: c.224C>T; p.(Thr75Met). This finding was subsequently validated through trio-based parental segregation analysis. The proband has two daughters. The 9-year-old daughter turned out to be genotype-positive. The child presented short stature, dysmorphic facial features and a neurodevelopmental disorder with pending specific functional assessment.

Regarding Patient 14, due to the nature of an external referral and specific longitudinal details concerning the sequential-versus-combination antiarrhythmic drug regimen, long-term post-ICD clinical outcomes were unavailable.

### 2.7. Family 7

The index case is a 13-years old Italian girl with an incidental finding of PVCs during a routine cardiac screening for competitive sports. She had an uneventful perinatal history; her birth weight and length were 2.4 kg and 48 cm, respectively. She showed normal growth and reached appropriate developmental milestones. ECG showed bidirectional fascicular PVCs (right bundle branch block and left anterior hemiblock; right bundle branch block and left posterior hemiblock) with short runs of non-sustained VT and prominent U-wave with R-on-U phenomenon during PVCs. Echocardiography was normal. No symptoms were reported in clinical history. Physical examination showed a broad forehead, low hairline, low-set right ear, long palpebral fissures, enophthalmia, high palate, multiple dental caries, low-set right ear, micro-retrognathia, temporomandibular joint instability, hand brachydactyly and camptodactyly, fifth digit clinodactyly and sloping shoulders. At age 13, her height was 142 cm (<3rd percentile) (Figure 2B,C, Pt 16). She was prescribed Nadolol and Flecainide medication. A channelopathy NGS custom panel screening identified the de novo pathogenic variant *KCNJ2*: c.412G>A; p.(Glu138Lys), which was then robustly validated by trio-based parental testing.

### 2.8. Family 8

The patient is an Italian, second-born girl of non-consanguineous parents. The pregnancy was unremarkable. She was born at 36 + 1 weeks’ gestation with a birth weight of 1.720 kg, a length of 43 cm, and an OFC of 29 cm. Physical examination showed proptosis, cleft palate, micrognathia, and short 4th and 5th metatarsals.

Her mother (who was present at genetic counseling) is affected by ATS with a genotype positive for the likely pathogenic variant *KCNJ2*: c.914C>T; p.(Thr305Ile). The mother has normal stature, cleft palate, micrognathia, scoliosis, and long QT interval. She had normal psychomotor development. The elder sister is also genotype-positive, but her clinical phenotype was not available. For this reason, she is not included in Table 1.

## 3. Results

This study includes 18 patients from eight unrelated families; 14 patients (77.8%) were females and 4 were males (22.2%).

The clinical presentations that prompted medical investigations in the probands included syncope (37.5%, *n* = 3/8), suspected LQTS (12.5%, *n* = 1) related to skeletal abnormalities, incidentally diagnosed asymptomatic PVCs (37.5%, *n* = 3) and short stature (12.5%, *n* = 1).

As detailed in the family case histories, the first detection of these cardiac signs consistently occurred during late childhood or adolescence (ranging from 8 to 13 years of age), further supporting the age-related penetrance discussed later.

In our cohort the ATS phenotype was extremely variable. The classical clinical triad of “heart–muscle–skeleton/craniofacial” manifestations was detected in a lower percentage compared to previously published data: 11.1% (*n* = 2/18) versus the literature report of 42.9–60%.

At the cardiac level, the presence of a prominent U-wave is the most common finding (90%, *n* = 9/10; data not available in 8). A long QT interval was diagnosed in 31.2% (5 out of 16 patients since the data was missing in 2); it was suspected in one patient and could not be calculated in another due to ventricular bigeminy (patient 7). PVCs were reported in seven cases (58.3%, *n* = 7/12; not available in 5). Seven patients (41.2%, *n* = 7/17; not available in 1) showed VT and three patients showed bigeminy. Most patients (82.3%, *n* = 14) were asymptomatic. Syncope was reported in four cases (22.2%); two of these patients underwent ICD implantation for recurrent syncope. Most patients started a specific therapy based on beta-blockers, and five patients received a combined treatment of beta-blockers and flecainide.

Peculiar facial features were consistently present (100%, *n* = 16/16; not available in 2), particularly a triangular face and low-set ears. Skeletal manifestations were highly prevalent, including digit defects such as brachydactyly (93.7%, *n* = 15/16; not available in 2), clinodactyly (81.2%, *n* = 13/16; not available in 2), variable syndactyly (37.5%, *n* = 6/16; not available in 2) and scoliosis (50%, *n* = 8/16; not available in 2). Twelve patients (66.6%, *n* = 12/18) showed short stature. Joint laxity was observed in 35.3% (*n* = 6/17; not available in 1) of cases.

The muscular phenotype was less prominent in our cohort, with only two patients (11.1%) presenting a history of periodic paralysis (PP) or muscle weakness. In one of these cases, PP was triggered by fluctuations in potassium levels. Because clinical management took place within dedicated multidisciplinary clinics, patients underwent both comprehensive neuromuscular evaluation and thorough endocrinological assessments to rule out secondary causes of electrolyte imbalances, which gave negative results. These findings suggest that the fluctuations were related to the ATS genotype. Due to persistent symptoms in the adolescent boy from Family 1, preventive treatment with acetazolamide was initiated and found to be well-tolerated; close monitoring of serum potassium levels was routinely performed to mitigate the drug’s potential kaliuretic effects. Neurodevelopmental disturbances, including variable intellectual disability and learning difficulties, were observed in four patients (23.5%, *n* = 4/17; not available in 1). None of the cohort showed moderate nor profound intellectual disability. No major psychiatric or major behavioral disturbances were observed.

## 4. Discussion

Andersen–Tawil Syndrome (ATS) is an ultra-rare genetic disorder with high intra and inter-familial variability. ATS is characteristically described by the triad of cardio-musculoskeletal involvement.

In 50–60% of cases, the condition is related to deleterious variants in *KCNJ2* encoding the α subunit of the K+ channel protein Kir2.1 that plays a relevant role in the regulation of heart rhythm by stabilizing the resting membrane potential and regulating the cell excitability [11]. A reasonable percentage of patients can still be genetically elusive.

The clinical presentation is characterized by incomplete, potentially age-related, penetrance of any of the following manifestations: **cardiac manifestations** as prominent U-waves, prolonged QTc, bidirectional VT, PVCs, and, rarely, polymorphic VT or torsade de pointes; **muscular manifestations** such as episodic flaccid muscle weakness, painful symptoms and PP; **distinctive facial features** such as broad forehead, triangular face, maxillary and mandibular hypoplasia, low-set ears, short palpebral fissures, wide nasal bridge, bulbous nose, thin upper lip, arched palate, **oligodontia and dental crowding**; **skeletal abnormalities,** including hand and foot defects such as fifth finger clinodactyly, second–third toe syndactyly, brachydactyly; and other skeletal abnormalities such as scoliosis, short stature, and joint laxity.

Regarding toe syndactyly, in our patients’ cohort, it was partial but bilateral in the majority of individuals. While partial toe syndactyly is known to be a relatively common finding within the general population, its clinical significance—as with many semiological features—depends on its clinical context. In isolation, partial toe syndactyly in an otherwise phenotypically normal individual may hold little diagnostic weight; however, when presenting within a constellation of other symptoms and features, it becomes a valuable component of a comprehensive diagnostic scoring system.

Notably, in our cohort, we found that the majority of affected individuals did not present with the classic clinical triad, which was reported in only a few patients (11.1%). This finding appears to be extremely low compared to previous literature data (42.9–60%).

The referring physician was not a cardiologist in 4 out of 8 families, representing 50% of the cohort. Specifically, the referral physician was an endocrinologist for short stature (1 family), a medical geneticist for dysmorphic features (2 families), and a sports medicine physician for asymptomatic PVCs (1 family).

In our cohort, most patients were asymptomatic from a cardiac perspective, and the majority were diagnosed subsequently to familial screening.

Prominent U-waves were detected in the majority of patients (90%). This finding is well-documented in the literature and has been suggested to be a pathognomonic sign for ATS [12,13].

In our cohort, LQTS was not a common finding (31.2%). Therefore, the presence of LQTS should not be considered a crucial sign to establish an ATS diagnosis, as also reported by Mazzanti et al. [12].

Seven patients showed PVCs, and all of these individuals had runs of VT. Five of them were adults, one was a 16-year-old patient, and another was aged 13.

Syncope was reported in four individuals (three adults and one at 16 years old). The latter also experienced VT, consistent with findings recently reported by Feng et al. [14].

Despite the heterogeneity of our cohort, these data might suggest that ATS exhibits age-related penetrance, with a higher risk of major arrhythmic events in adulthood than in childhood.

Muscular involvement was the least represented manifestation in our cohort and underlies the low percentage of cases manifesting the classic ATS triad. These data are probably related to the fact that muscular symptoms, such as weakness, can often be underestimated by patients themselves.

Skeletal features, including overall hands and feet abnormalities and short stature, were also prevalent in our cohort and they may assist in the diagnostic approach. In particular, brachydactyly and fifth digit clinodactyly were prominent (93.7% and 81.2%, respectively). However, we suggest careful physical examination for other underestimated characteristics such as scoliosis (50%), joint laxity (35.3%) or dental anomalies (46.1%).

Distinctive facial features were observed in all directly observed individuals (100%). We find this element extremely relevant in the diagnostic process of ATS patients. In fact, a rightful evaluation by an expert clinical geneticist may lead to an early clinical diagnosis, even before the onset of cardiac or muscular manifestations. However, it is important to note that our cohort exclusively includes patients carrying *KCNJ2* variants (ATS Type 1). Since *KCNJ5*-related ATS (Type 2) accounts for approximately 15% of cases and may exhibit a different facial phenotype, further studies are required to determine whether these diagnostic considerations can be fully extended to Type 2 patients. For this reason, in this paper, we might propose the integration of facial features as a further diagnostic criteria and suggest the term cardio-musculoskeletal–facial syndrome.

Furthermore, the specificity of these features must be carefully evaluated to reliably differentiate ATS from clinical mimics with overlapping dysmorphic, stature, cognitive, or dental anomalies, such as RASopathies or chromatin remodeling and related epigenetic disorders (including KBG and Coffin–Siris syndromes). The latter constellations carry short stature and dental features, but their cardiac phenotype is fundamentally distinct from the ATS context.

Neurocognitive assessment should also be investigated in suspected ATS considering possible intellectual disabilities or learning difficulties.

## 5. Limitations

Our cohort’s notable female predominance (77.8%; 14/18 patients) contrasts with the expected equal sex distribution of an autosomal dominant disorder like ATS. A similar recent observation was described in which females with ATS were more prone to manifestations related to ATS [6]. In that meta-analysis, there was compelling evidence for a pronounced gender disparity among inherited QT-prolonging arrhythmias, with ATS females demonstrating exceptional vulnerability to life-threatening events. While this indicates a distinct biological susceptibility to cardiac manifestations, the overrepresentation of females in our specific cohort may also be independently influenced by an ascertainment or referral bias. We hypothesize that extra-cardiac triad signs, such as distinctive facial features and short stature, might be clinically more accentuated or prioritized in females, thereby more readily prompting clinical referral in girls than in boys within specialized networks. Acknowledging this selective referral bias provides critical context for interpreting our finding that distinctive facial features were constant (100%) in the studied population

## 6. Conclusions

In the management of the ATS population, it is important to consider not only the major signs classically associated with the disease such as arrhythmic spectrum disorders (i.e., QTc prolongation) but also other multisystemic aspects that may be underestimated despite having a significant impact on patients’ quality of life. In half of our cohort, the presenting sign or symptom was not identified by a cardiologist, as cardiac manifestations can often be subtle. Consequently, the first referring physician may be a pediatrician, an endocrinologist, or a clinical geneticist. Therefore, we underline the importance of a multidisciplinary, personalized, and longitudinal approach to the management of ATS.

Based on our findings, we propose the integration of distinctive facial features as an additional diagnostic criterion, given that this phenotype was consistently present (100%) in our cohort. While we acknowledge our sample size limits a formal renaming into cardio-musculoskeletal–facial syndrome, we instead propose that characteristic facial features when read by physicians trained in the cardiogenetic field be considered an additional diagnostic criterion. To objectify this facial gestalt, future work should incorporate automated evaluations like Face2Gene similarity scores (https://www.face2gene.com/, accessed on 8 January 2026) [15] or DeepGestalt v21.5.0 match rates to standardize assessment. Furthermore, the specificity of these features must be carefully evaluated to reliably differentiate ATS from clinical mimics with overlapping dysmorphic, stature, cognitive or dental anomalies. Further studies on multicentric basis with deep multidisciplinary phenotyping are needed to further delineate this rare condition.

## Figures and Tables

**Table 1:** Genotyping and phenotyping data of our cohort including cardiac and extra-cardiac features.

	Pt 1	Pt 2	Pt 3	Pt 4	Pt 5	Pt 6	Pt 7	Pt 8	Pt 9	P 10	Pt 11	Pt 12	Pt 13	Pt 14	Pt 15	Pt 16	P 17	P 18	
Family	1	1	1	1	2	2	3	3	3	4	4	5	5	6	6	7	8	8	
M/F	M	F	F	F	F	F	F	F	F	M	M	M	F	F	F	F	F	F	F 77.8%; M 22.2%
Age at last observation (years)	14	12	46	44	17	14	19	23	54	20	50	16	33	36	9	13	0	37	Mean age: 25.4; median age: 19.5 (IQR: 14.0–37.0 years, range 0–54)
*KCNJ2* genotype	c.244C>T p.(Arg82Trp)	c.244C>T p.(Arg82Trp)	c.244C>T p.(Arg82Trp)	c.244C>T p.(Arg82Trp)	c.244C>T p.(Arg82Trp)	c.244C>T p.(Arg82Trp)	c.13C>T p.(Arg5Ter)	c.13C>T p.(Arg5Ter	c.13C>T p.(Arg5Ter)	c.245G>A p.(Arg82Gln)	c.245G>A p.(Arg82Gln)	c.251T>G p.(Met84Arg)	c.251T>G p.(Met84Arg)	c.224C>T p.(Thr75Met)	c.224C>T p.(Thr75Met)	c.412G>A p.(Glu138Lys)	c.914C>T p.(Thr305Ile)	c.914C>T p.(Thr305Ile)	
ACMG class variant	5	5	5	5	5	5	4	4	4	5	5	4	4	5	5	5	4	4	
Presenting sign or symptom	Family screening	Family screening	Syncope starting: 8y old	Family screening	Short stature	Short stature	PVCs at 13y old/ asymptomatic	Family screening	Family screening	PVCs at 12y old/ asymptomatic	Family screening	Syncope and dysmorphic features	Family screening	Syncope	Family screening	PVC-asymptomatic/ asymptomatic	Family screening	Long QT and skeletal abnormalities	
Syncope	No	No	Yes	Yes	No	No	No	No	No	No	No	Yes	No	Yes	No	No	No	No	4/18 22.2
QTc (ms)	Normal	Normal	470–480	Normal	Normal	Normal	Not calculated due to bigeminy	Normal	Normal	490	Normal	490	N.A.	500	Normal	Normal	Suspected LQT	LQT (undefined)	5/16 31.2% 1 suspected 1 not calculated
Prominent U-waves	Yes	Yes	Yes	No	Yes	Yes	Yes	N.A.	N.A.	Yes	N.A.	Yes	N.A.	N.A.	N.A.	Yes	N.A.	N.A.	9/10 (90%)
PVCs	No	No	Yes	No	No	No	Yes	N.A.	N.A.	Yes	N.A.	Yes	Yes	Yes	N.A.	Yes	N.A.	N.A.	7/12 58.3%
VT	No	No	Yes	No	No	No	Yes	No	No	Yes	No	Yes	Yes	Yes	No	Yes	No	N.A.	7/17 (41.2%)
ICD	No	No	Yes	No	No	No	No	No	No	No	No	No	No	Yes	No	No	No	N.A.	2/17 (11.8%)
Therapy	Nadolol	Nadolol	Bisoprolol	Bisoprolol	Nadolol	Nadolol.	Nadolol. Flecainide	N.A.	N.A.	Nadolol. Flecainide	N.A.	Atenolol. Flecainide	Amiodarone	Propranolol. Flecainide	N.A.	Nadolol. Flecainide	N.A.	N.A.	
Facial features	Yes	Yes	Yes	Yes	Yes	Yes	Yes	Yes	Yes	N.A.	N.A.	Yes	Yes	Yes	Yes	Yes	Yes	Yes	16/16 100%
Short stature	No	No	Yes	Yes	Yes	Yes	Yes	Yes	Yes	No	No	No	Yes	Yes	Yes	Yes	Yes	No	12/18 66.6%
Clinodactyly	Yes	Yes	Yes	Yes	Yes	Yes	Yes	Yes	Yes	N.A.	N.A.	No	Yes	Yes	Yes	Yes	No	No	13/16 81.2%
Syndactyly	Yes	Yes	No	No	Yes	Yes	Yes	No	Yes	N.A.	N.A.	No	No	No	No	No	No	No	6/16 37.5%
Brachydactyly	Yes	Yes	Yes	Yes	Yes	Yes	Yes	Yes	Yes	N.A.	N.A.	Yes	Yes	Yes	Yes	Yes	Yes	No	15/16 93.7%
Scoliosis	Yes	Yes	Yes	Yes	Yes	Yes	No	No	No	Yes (also *pectus excavatum*)	N.A.	No	No	No	No	No	N.A.*	Yes	8/16 50%
Joint laxity	No	No	Yes	No	Yes	Yes	No	No	No	No	N.A.	No	No	Yes	Yes	Yes	N.A.*	No	6/16 37.5%
Dental anomalies	No	No	No	No	Yes	Yes	Yes	No	No	N.A.	N.A.	Yes	Yes	N.A.	N.A.	Yes	N.A.*	No	6/13 46.1%
Periodic paralysis/ muscle weakness	Yes	Yes	No	No	No	No	No	No	No	No	No	No	No	No	No	No	N.A.*	No	2/17 11.7%
Intellectual disability	No	No	No	No	Yes	Yes	No	No	No	No	No	No	No	Yes	Yes	No.	N.A.*	No	4/17 23.5%

Abbreviations: ACMG, American College of Medical Genetics; ICD, implantable cardioverter defibrillator; N.A., not available; PVC, premature ventricular contraction; VT, ventricular tachycardia; * data excluded from the analysis because they were not assessable due to the patient’s age.

## Data Availability

The original contributions presented in this study are included in the article. Further inquiries can be directed to the corresponding author.
